# Author Correction: The interrelation of sleep and mental and physical health is anchored in grey-matter neuroanatomy and under genetic control

**DOI:** 10.1038/s42003-020-1017-y

**Published:** 2020-06-02

**Authors:** Masoud Tahmasian, Fateme Samea, Habibolah Khazaie, Mojtaba Zarei, Shahrzad Kharabian Masouleh, Felix Hoffstaedter, Julia Camilleri, Peter Kochunov, B. T. Thomas Yeo, Simon Bodo Eickhoff, Sofie Louise Valk

**Affiliations:** 1 Institute of Medical Science and Technology, Shahid Beheshti University, Tehran, Iran; 2 Sleep Disorders Research Center, Kermanshah University of Medical Sciences, Kermanshah, Iran; 3 Institute of Neuroscience and Medicine (INM-7: Brain and Behaviour), Research Centre Jülich, 52425 Jülich, Germany; 4 Institute of Systems Neuroscience, Heinrich Heine University Düsseldorf, 40225 Düsseldorf, Germany; 5 Maryland Psychiatric Research Center, University of Maryland School of Medicine, Baltimore, MD 21201 USA; 6 Department of Electrical and Computer Engineering, Clinical Imaging Research Centre, N.1 Institute for Health and Memory Networks Program, National University of Singapore, Singapore, 119077 Singapore; 7 Athinoula A. Martinos Center for Biomedical Imaging, Massachusetts General Hospital, Charlestown, MA 02114 USA; 8 Centre for Sleep and Cognition, National University of Singapore, Singapore, 119077 Singapore; 9 NUS Graduate School for Integrative Sciences and Engineering, National University of Singapore, Singapore, 119077 Singapore

**Keywords:** Sleep, Genetics of the nervous system, Circadian rhythms and sleep

Correction to: *Communications Biology* 10.1038/s42003-020-0892-6, published online 9 April 2020.

In the original published version of the article, the Methods section entitled “Statistics and reproducibility” incorrectly stated that the coefficient of relationship between individuals in the HCP sample was computed using the KING method. However, the coefficient of relationship was computed using self-reported twin status data. The error has been corrected in the HTML and PDF versions of the paper.

