# Supplementary material for: The interrelation of sleep and mental and physical health is anchored in grey-matter neuroanatomy and under genetic control
Source: Commun Biol. 2020 Apr 9;3:171. doi: 10.1038/s42003-020-0892-6 (PMC7145855; doi:10.1038/s42003-020-0892-6)
Supplement: Supplementary file 1 — Supplementary Information [file 42003_2020_892_MOESM1_ESM.pdf]

PHENOTYPIC AND GENETIC CORRELATION BETWEEN SLEEP, BEHAVIOR, AND MACROSCALE CORTICAL GREY MATTER

SUPPLEMENT

SUPPLEMENTARY FIGURES

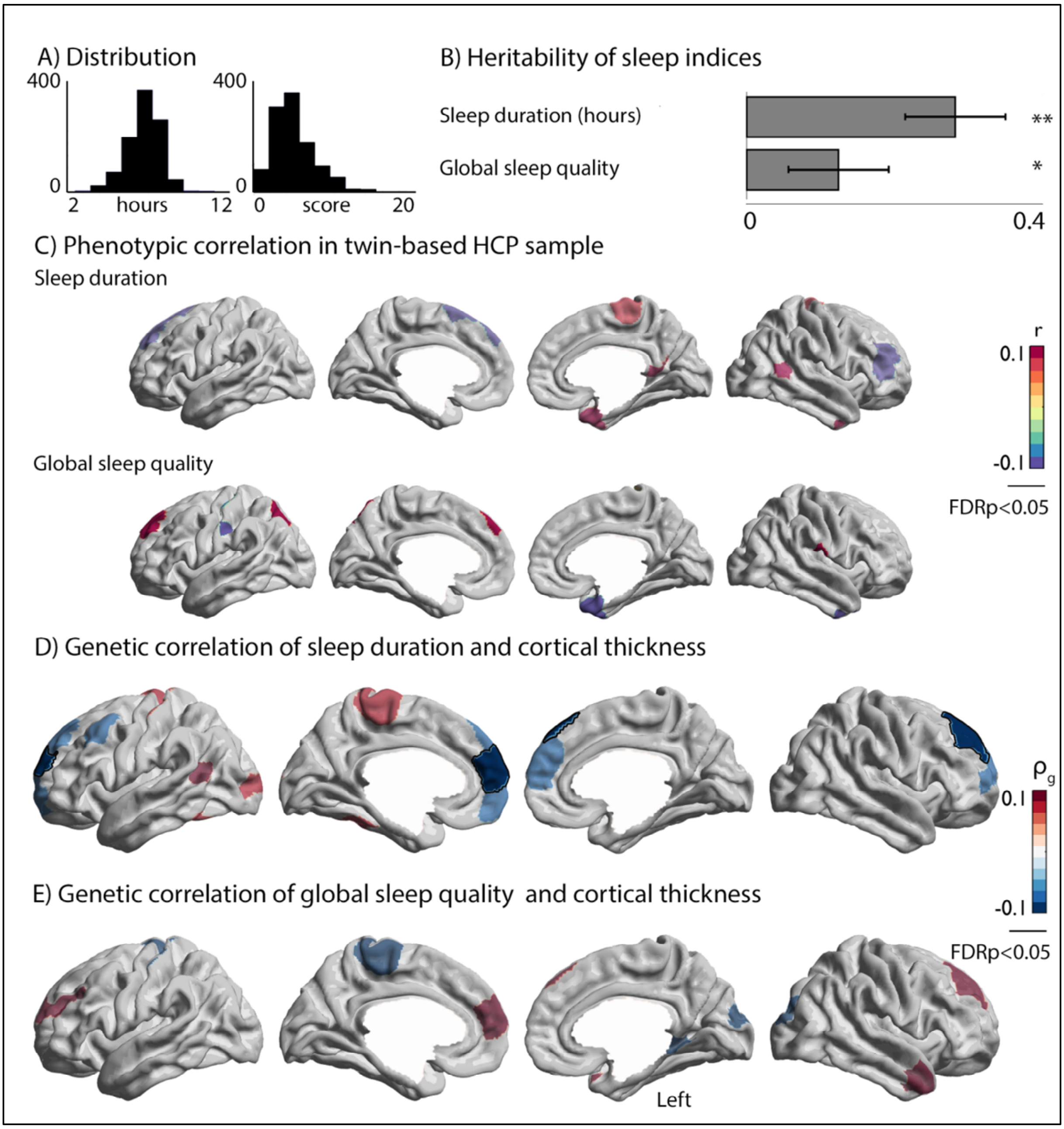

Supplementary Fig 1. Shared genetic basis of sleep duration and cortical thickness. *A) Distribution of variables; B) Heritability of sleep duration and global sleep quality score. Significant FDR correction (p<0.05/2) heritability is annotated with **, and p<0.05 with *; C). Phenotypic correlation of sleep duration and global sleep quality in the related sample; D+E) genetic correlation of sleep duration/global sleep quality and voxel-wise cortical thickness. Significant associations (FDRq<0.05/2) between sleep indices and brain structure have black outline, whereas trends (p<0.01) are transparent. Red indicates a positive relationship, whereas blue indicates a negative genetic relationship between sleep and brain structure.*

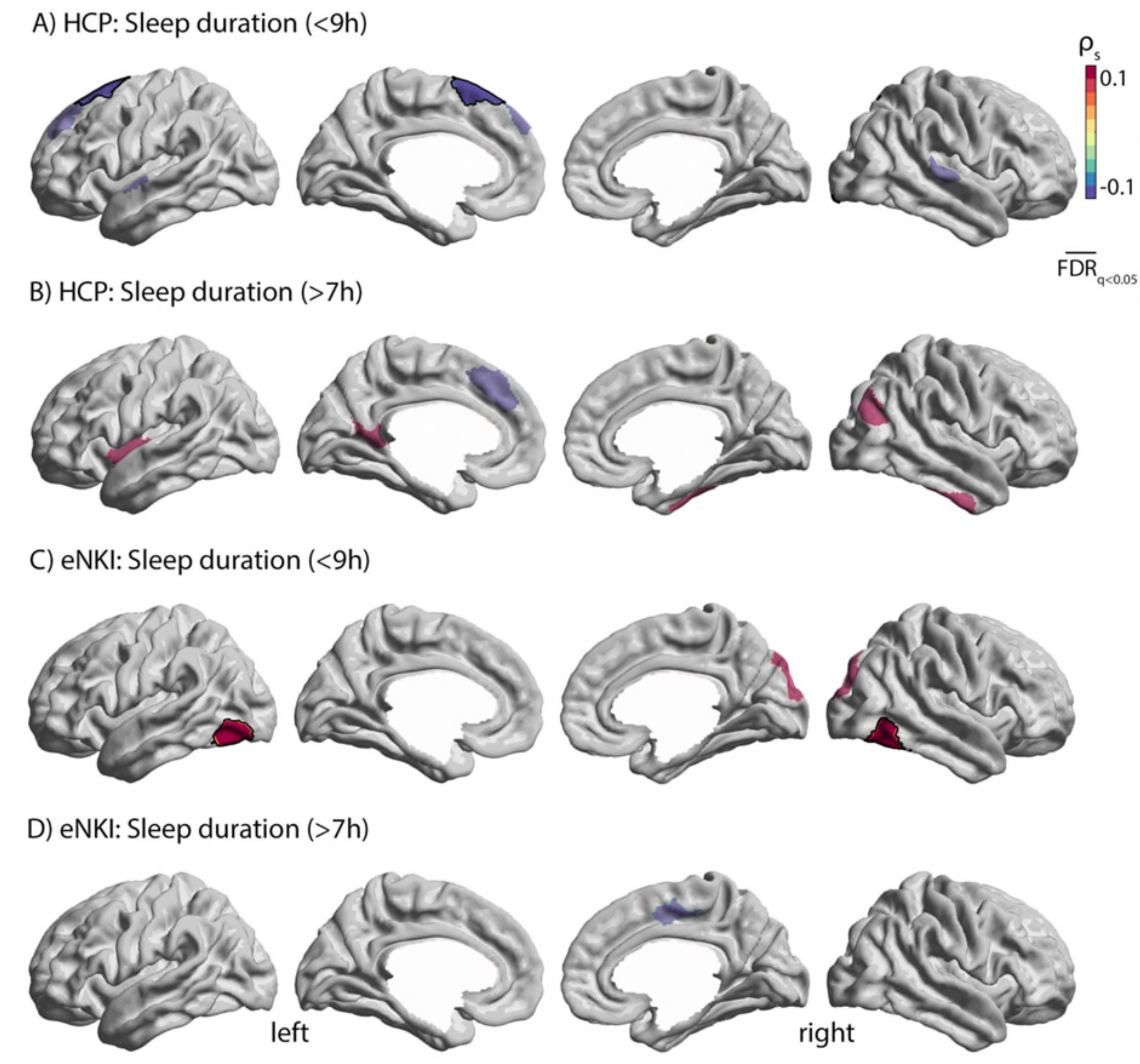

Supplementary Fig 2. Relationship between short and long sleep duration and brain structure. *A) Linear relationship between short to normal sleep (<9h) and local cortical thickness in the unrelated HCP subsample; B) Linear relationship between normal to long sleep (>=7h) and local cortical thickness in the unrelated HCP subsample; C) Linear relationship between short to normal sleep (<9h) and local cortical thickness in the eNKI sample; D) Linear relationship between normal to long sleep (>=7h) and local cortical thickness in the eNKI sample. Significant associations (FDR q<0.05) between sleep indices and brain structure have black outline, whereas trends (p<0.01) are transparent. Red indicates a positive relationship, whereas blue indicates a negative genetic relationship between sleep and brain structure.*

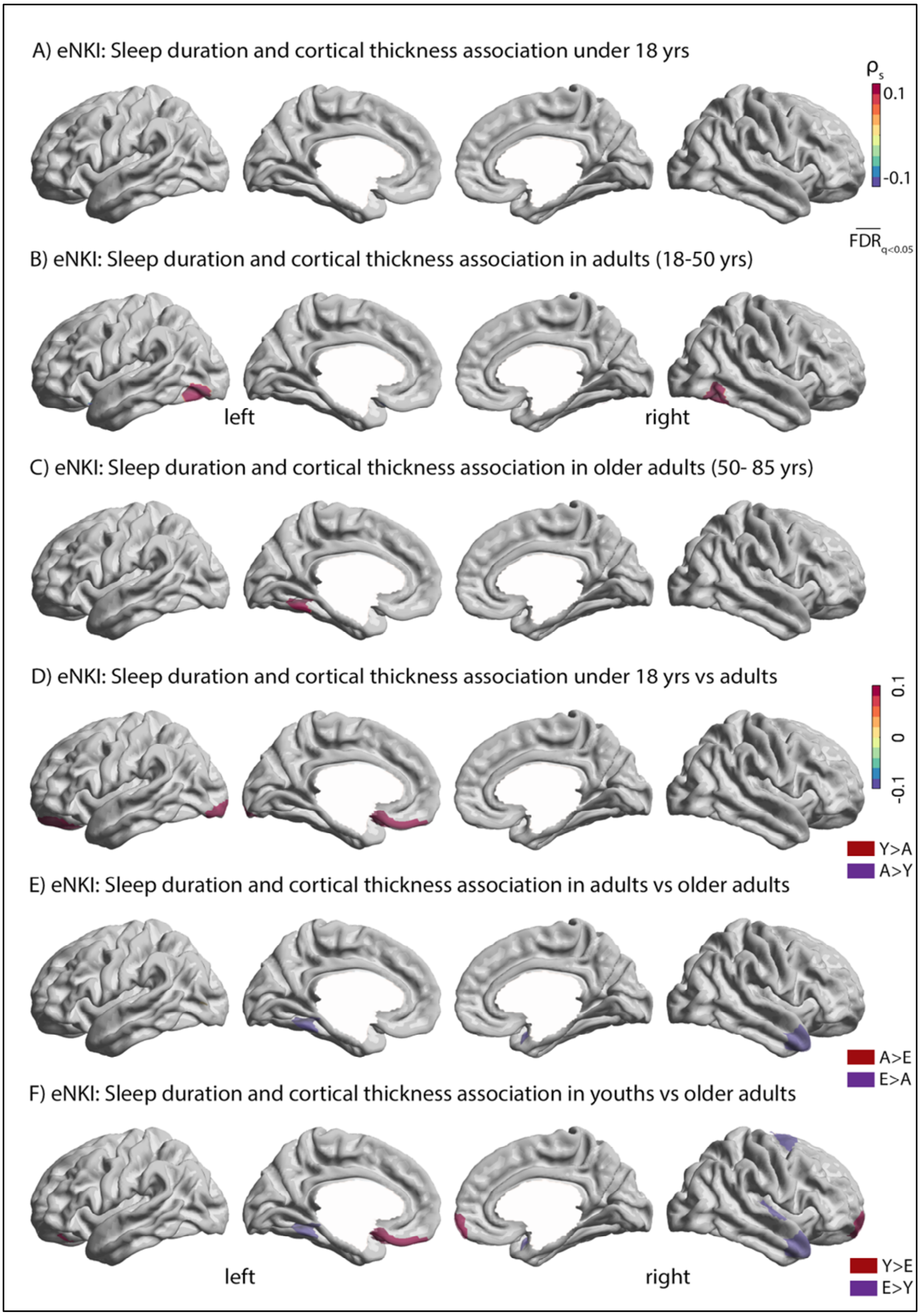

Supplementary Fig 3. Age-specific modulation of the relation between sleep duration and cortical thickness. *In the eNKI sample, we split the sample in youths (12-17), adults (18-49), and elderly individuals (50-85) and evaluated the relationship between thickness and sleep duration in each of these age-group separately as well as compared whether effects of sleep duration on brain structure were different between groups. Findings at FDRq<0.05 have a black outline, and trends at p<0.01 are reported at 60% transparency.*

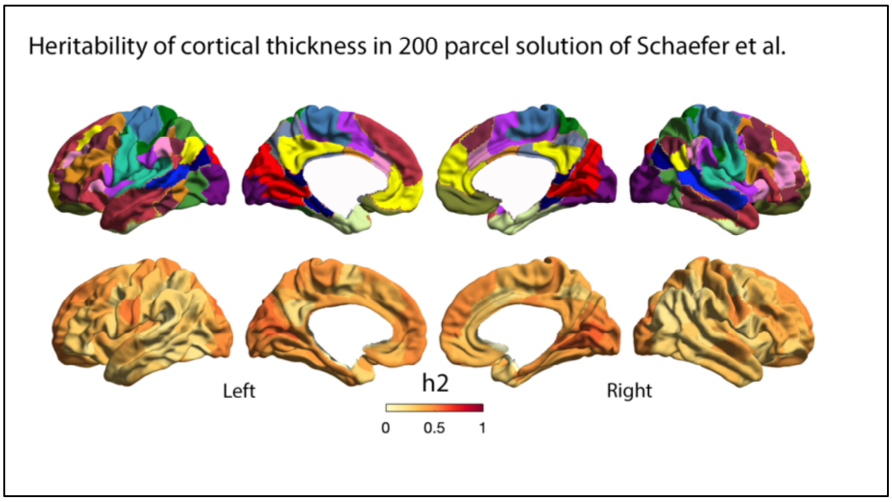

Supplementary Fig 4. Heritability of cortical thickness. *Heritability of brain structure (lower panel) in the 200-parcel solution of Schaefer atlas*[1] *(upper panel). The 200-parcel solution by Schaefer et al is based on both local and global functional connectivity. In each parcel, we extracted trimmed mean (10%) cortical thickness values of each individual. Subsequently, we computed heritability of variance in cortical thickness in each region and displayed this on the cortex. Please see Supplementary Table 9 for heritability values as well as standard errors and significant level (p-values).Notably all regions but the*

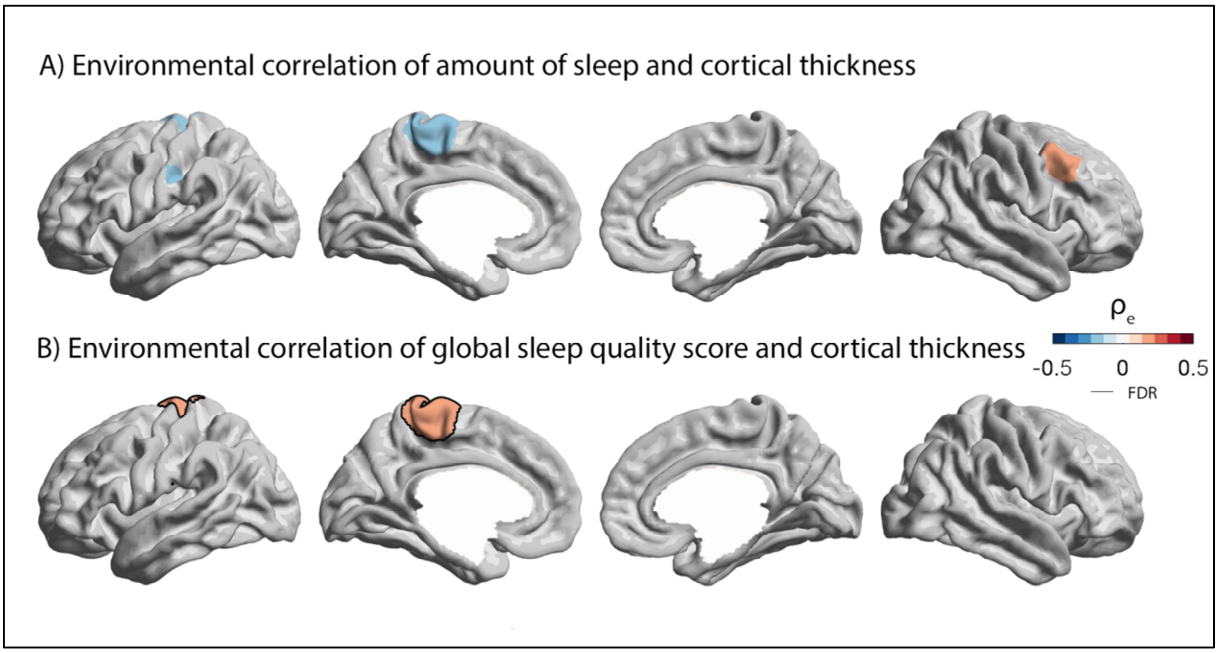

Supplementary Fig 5. Environmental relationship between sleep and brain structure. *A) Environmental correlation between sleep duration and brain structure and B) global sleep quality and brain structure. Significant associations between sleep indices and brain structure have black outline, whereas trends (p<0.01) are transparent. Red indicates a positive relationship, whereas blue indicates a negative genetic relationship between sleep and brain structure.*

Functional decoding of neurogenetic markers of sleep

To enable inference about functional brain processes that are sustained by the regions showing significant phenotypic and genetic correlation with sleep duration, we performed quantitative functional decoding using the BrainMap database[2]. Here, we observed the left superior frontal cortex, phenotypically implicated in sleep duration in the HCP unrelated sample, to be activated by tasks involving (socio-) cognitive, action, and emotion processes ($p<0.05$). In the eNKI sample we observed phenotypic correlation in bilateral inferior temporal regions as well as right posterior occipital cortex, which were all associated with action observation, perception, as well as spatial cognition ($p<0.05$). In addition, inferior temporal regions were associated with spatial cognition as well as negative affect and interoception ($p<0.05$). Last, the left medial frontal cortex and right superior frontal cortex, which had genetic correlation with sleep duration, were implicated in processes involving (socio)-cognition and emotion (Supplementary Fig 6).

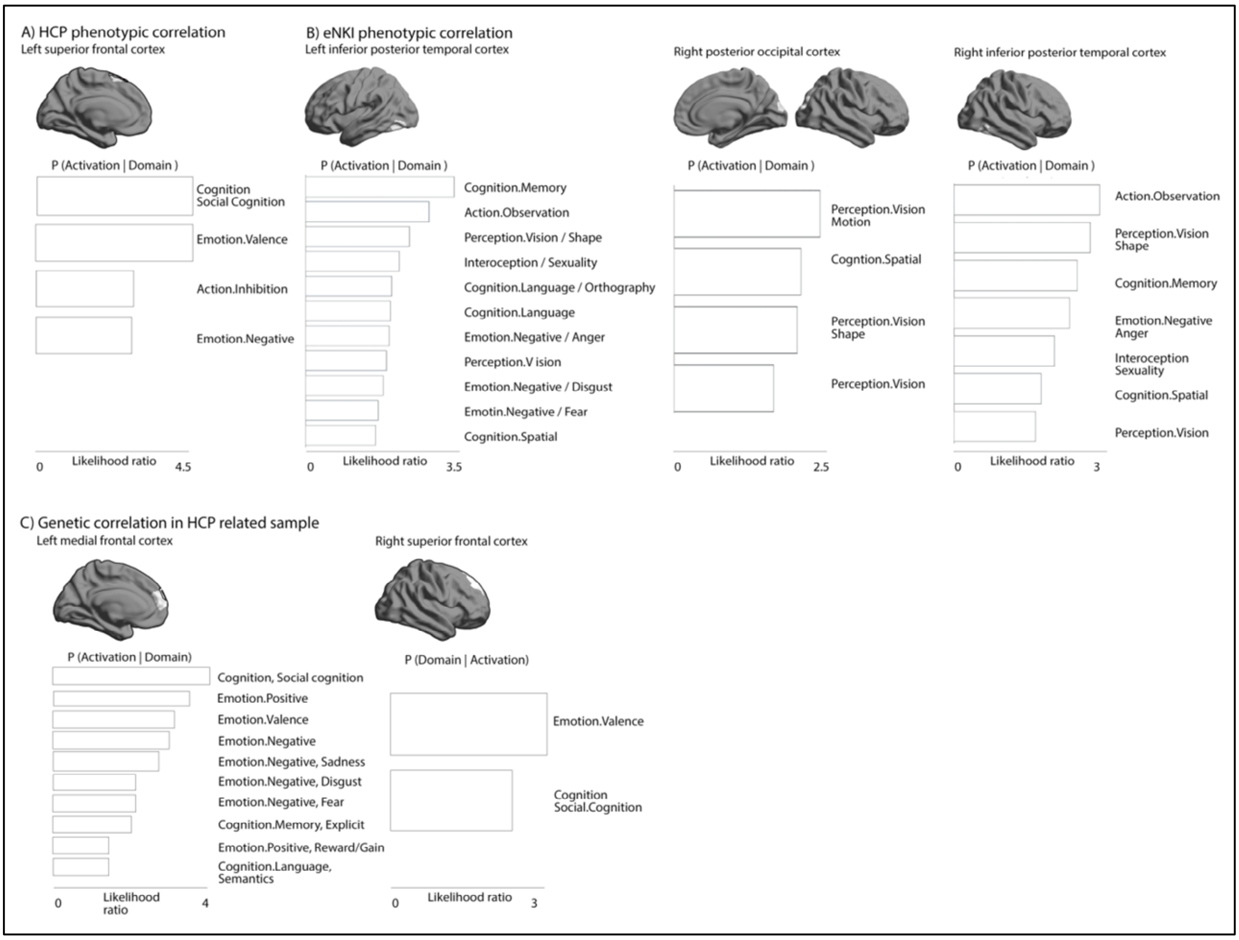

Supplementary Fig 6. Cortical thickness correlates of sleep duration are functionally linked with cognitive and emotion processing. *Quantitative functional decomposition of brain markers of sleep based on the FDR-corrected clusters of phenotypical/genetic relationships shown in Fig 1 and Fig S1.*

SUPPLEMENTARY TABLE S

CogTotalComp_Unadj	DSM_Depr_Pct
CogFluidComp_Unadj	DSM_Avoid_Pct
CogCrystalComp_Unadj	DSM_Adh_Pct
ReadEng_Unadj	Weight
PicVocab_Unadj	DSM_Inat_Raw
ListSort_Unadj	DSM_Anxi_Pct
CardSort_Unadj	BMI
ProcSpeed_Unadj	SSAGA_Depressive_Sx
Flanker_Unadj	DSM_Hype_Raw
Flanker_AgeAdj	SSAGA_Educ
PicSeq_Unadj	SSAGA_BMICat
IWRD_TOT	DSM_Antis_Raw
NEOFAC_O	DSM_Somp_Pct
DDisc_AUC_40K	SSAGA_Depressive_Ep
MMSE_Score	SSAGA_ChildhoodConduct
DDisc_AUC_200	SSAGA_PanicDisorder
LifeSatisf_Unadj	SSAGA_Times_Used_Opiates
SelfEff_Unadj	SSAGA_Times_Used_Stimulants
NEOFAC_A	SSAGA_Times_Used_Illicits
InstruSupp_Unadj	SSAGA_Income
EmotSupp_Unadj	SSAGA_Times_Used_Hallucinogens
FearSomat_Unadj	SSAGA_TB_Yrs_Smoked
Friendship_Unadj	SSAGA_TB_DSM_Withdrawal
Loneliness_Unadj	SSAGA_Mj_Age_1st_Use
PSQI_Comp7	SSAGA_Times_Used_Sedatives
PosAffect_Unadj	SSAGA_InSchool
MeanPurp_Unadj	Height
Sadness_Unadj	SSAGA_Times_Used_Cocaine
AngAffect_Unadj	SSAGA_TB_Age_1st_Cig
NEOFAC_E	SSAGA_Mj_Use
FearAffect_Unadj	SSAGA_TB_Max_Cigs
PSQI_Comp6	SSAGA_Rlshp
PercHostil_Unadj	SSAGA_Agoraphobia
PSQI_Comp1	SSAGA_Mj_Ab_Dep
AngAggr_Unadj	SSAGA_Employ
PercReject_Unadj	SSAGA_Mj_Times_Used
AngHostil_Unadj	SSAGA_TB_Still_Smoking
PSQI_Comp4	SSAGA_TB_Smoking_History
NEOFAC_C	SSAGA_TB_Yrs_Since_Quit
NEOFAC_N	SSAGA_HSI_Score
PSQI_Comp5	SSAGA_TB_Hvy_CPD

PSQI_Comp2	SSAGA_FTND_Score
PercStress_Unadj	SSAGA_TB_DSM_Tolerance
PSQI_Score	SSAGA_TB_Reg_CPD
PSQI_Comp3	SSAGA_MOBorn
	SSAGA_TB_DSM_Difficulty_Quitting

Supplementary Table 1. List of selected behavior markers in HCP sample. *Behavioral markers to evaluate the relation between sleep and cognition, mental, and physical health. For further details, please see: https://wiki.humanconnectome.org/display/PublicData*

DDisc_AUC_40K	r=0.19	p=6.65e-11
NEOFAC_A	r=0.18	p=7.25e-10
DDisc_AUC_200	r=0.17	p=3.34e-09
LifeSatisf_Unadj	r=0.15	p=4.41e-07
ReadEng_Unadj	r=0.14	p=2.51e-06
CogCrystalComp_Unadj	r=0.13	p=3.67e-06
PicVocab_Unadj	r=0.12	p=5.22e-05
CogTotalComp_Unadj	r=0.10	p=7.93e-04
PosAffect_Unadj	r=0.09	p=1.64e-03
EmotSupp_Unadj	r=0.08	p=3.65e-03
PercHostil_Unadj	r=-0.06	p=2.88e-02
Sadness_Unadj	r=-0.07	p=1.36e-02
Loneliness_Unadj	r=-0.07	p=1.02e-02
PercReject_Unadj	r=-0.08	p=5.07e-03
AngHostil_Unadj	r=-0.09	p=1.16e-03
FearSomat_Unadj	r=-0.10	p=8.20e-04
NEOFAC_N	r=-0.10	p=6.00e-04
AngAffect_Unadj	r=-0.11	p=1.56e-04
PSQI_Comp2	r=-0.11	p=9.49e-05
PSQI_Comp5	r=-0.11	p=7.77e-05
PercStress_Unadj	r=-0.13	p=6.29e-06
PSQI_Comp7	r=-0.14	p=1.06e-06
PSQI_Comp1	r=-0.35	p=1.20e-36
PSQI_Comp4	r=-0.40	p=3.97e-47
PSQI_Score	r=-0.55	p=2.21e-95
PSQI_Comp3	r=-0.89	p=00
SSAGA_Educ	r=0.13	p=6.20e-06
SSAGA_Times_Used_Hallucinogens	r=0.08	p=5.05e-03
SSAGA_Income	r=0.07	p=9.89e-03
DSM_Hype_Raw	r=-0.08	p=4.32e-03
Weight	r=-0.08	p=3.94e-03
DSM_Depr_Pct	r=-0.09	p=1.68e-03
DSM_Anxi_Pct	r=-0.11	p=1.53e-04
BMI	r=-0.11	p=7.44e-05

SSAGA_BMICat	r=-0.11	p=7.18e-05
DSM_Antis_Raw	r=-0.12	p=1.89e-05

Supplementary Table 2. Phenotypic association between sleep duration and behavior in full HCP sample. *Spearman's rho between sleep duration and the respective behavioral score in the full HCP sample. Only associations surviving FDRq<0.05 corrections are reported.*

PSQI_Score	r=1.00	p=00
PSQI_Comp1	r=0.67	p=4.80e-157
PSQI_Comp2	r=0.59	p=5.80e-113
PSQI_Comp3	r=0.58	p=8.69e-108
PSQI_Comp4	r=0.57	p=6.76e-105
PSQI_Comp5	r=0.50	p=9.63e-76
PSQI_Comp7	r=0.46	p=4.10e-63
PSQI_Comp6	r=0.34	p=7.90e-35
PercStress_Unadj	r=0.30	p=3.25e-27
AngAffect_Unadj	r=0.30	p=3.65e-26
NEOFAC_N	r=0.26	p=1.62e-20
FearAffect_Unadj	r=0.26	p=1.46e-19
Sadness_Unadj	r=0.24	p=3.68e-17
FearSomat_Unadj	r=0.23	p=2.05e-16
Loneliness_Unadj	r=0.21	p=5.72e-14
AngHostil_Unadj	r=0.21	p=1.22e-13
PercReject_Unadj	r=0.21	p=3.46e-13
AngAggr_Unadj	r=0.15	p=1.87e-07
PercHostil_Unadj	r=0.15	p=2.87e-07
NEOFAC_O	r=0.02	p=4.03e-01
ListSort_Unadj	r=-0.03	p=2.74e-01
Flanker_Unadj	r=-0.03	p=2.68e-01
Flanker_AgeAdj	r=-0.04	p=2.22e-01
ProcSpeed_Unadj	r=-0.04	p=1.70e-01
PicSeq_Unadj	r=-0.04	p=1.66e-01
CardSort_Unadj	r=-0.04	p=1.47e-01
CogFluidComp_Unadj	r=-0.05	p=7.95e-02
IWRD_TOT	r=-0.06	p=3.52e-02
SelfEff_Unadj	r=-0.08	p=4.72e-03
MMSE_Score	r=-0.09	p=2.60e-03
CogTotalComp_Unadj	r=-0.10	p=4.60e-04
PicVocab_Unadj	r=-0.12	p=2.94e-05
InstruSupp_Unadj	r=-0.12	p=2.79e-05
NEOFAC_C	r=-0.12	p=1.99e-05
ReadEng_Unadj	r=-0.13	p=1.09e-05
NEOFAC_E	r=-0.13	p=5.04e-06
CogCrystalComp_Unadj	r=-0.13	p=4.45e-06

DDisc_AUC_40K	r=-0.16	p=1.02e-08
Friendship_Unadj	r=-0.16	p=8.90e-09
DDisc_AUC_200	r=-0.17	p=4.32e-09
MeanPurp_Unadj	r=-0.17	p=1.39e-09
EmotSupp_Unadj	r=-0.18	p=3.52e-10
PosAffect_Unadj	r=-0.20	p=5.09e-12
NEOFAC_A	r=-0.21	p=9.82e-14
LifeSatisf_Unadj	r=-0.25	p=3.10e-19
SSAGA_Educ	r=0.13	p=6.20e-06
SSAGA_Times_Used_Hallucinogens	r=0.08	p=5.05e-03
SSAGA_Income	r=0.07	p=9.89e-03
DSM_Hype_Raw	r=-0.08	p=4.32e-03
Weight	r=-0.08	p=3.94e-03
DSM_Depr_Pct	r=-0.09	p=1.68e-03
DSM_Anxi_Pct	r=-0.11	p=1.53e-04
BMI	r=-0.11	p=7.44e-05
SSAGA_BMICat	r=-0.11	p=7.18e-05
DSM_Antis_Raw	r=-0.12	p=1.89e-05

Supplementary Table 3 Phenotypic association between global sleep quality and behavior in the full HCP sample. *Spearman's rho between sleep quality and the respective behavioral score in the full HCP sample. Only associations surviving FDRq<0.05 corrections are reported (corrected for number of behavioral markers).*

**Interrelations HCP all (n=1113)**			
	Depression	BMI	IQ
Depression	1		
BMI	0.05, p>0.2	1	
IQ	-0.02, p>0.3	-0.17, p<0.0001**	1
**Interrelations HCP unrelated subsample**			
	Depression	BMI	IQ
Depression	1		
BMI	0.21, p<0.0001**	1	
IQ	0.00, p>ns	-0.19, p<0.0001**	1
**Interrelations: eNKI**			
	Depression	BMI	IQ
Depression	1		
BMI	0.12, p<0.002**	1	
IQ	-0.11, p<0.002**	-0.15, p<0.0001**	1
**Interrelations: Genetic correlation** [3]			
	Depression	BMI	IQ
Depression	1		
BMI	-0.02 [0.12], p>ns	1	
IQ	0.02 [0.10], p>ns	-0.27 [0.06], p<0.0001**	1
**Interrelations: environmental correlation** [3]			
	Depression	BMI	IQ
Depression	1		
BMI	0.12 [0.07], p>ns	1	
IQ	-0.08 [0.24], p>ns	0.10 [0.07], p>ns	1

Supplementary Table 4. Phenotypic and genetic correlations between confounding effects on sleep (Depression, BMI, and IQ). *Exact p-values are reported, ** indicates FDRq<0.05, controlled for number of tests (6) and number of samples studied (3), * indicates trend at p<0.05.*

	HCP unrelated	*HCP complete*	*eNKI*
Short	279/424	750/1113	559/783
Normal	133/424	328/1113	155/783
Long	12/424	35/1113	69/783

Supplementary Table 5. Number of short, normal and long sleepers in the HCP and eNKI sample based on previous study[4] *. Short sleepers: 7h or shorter; normal sleepers: between 7 and 9h of sleep, long sleepers: 9h or longer.*

**eNKI**	**Sleep duration *m(sd) min - max***
Youth (12-17)	7.5(1.5) 4-12
Adult (18-49)	6.8(1.3) 3-11
Elderly (50-85)	6.7(1.2) 3-10

Supplementary Table 6. Sleep duration based on three age groups in the eNKI sample.

**Sleep duration**	**HCP (unrelated sample)**	**HCP (total sample)**	**eNKI**	**Genetic**
HCP (unrelated sample)	1			
HCP (total sample)	0.77 [0.69 0.83], p<0.00001**	1		
eNKI	0.30 [0.16 0.44], p<0.00001**	0.16 [0.02 0.29], p<0.00001**	1	
Genetic	0.53 [0.42 0.63], p<0.00001**	0.61 [0.51 0.69], p<0.00001**	0.24 [0.12 0.37], p<0.00001**	1
**Global sleep quality**	**HCP (unrelated sample)**	**HCP (total sample)**	**eNKI**	**Genetic**
HCP (unrelated sample)	1			
HCP (total sample)	0.64 [0.54 0.72], p<0.00001**	1		
eNKI	0.13 [-0.01 0.26] p>0.05	-0.09 [-0.22 0.06] p>0.1	1	
Genetic	0.19. [0.05. 0.32], p<0.0001**	0.48 [0.36 0.59], p<0.00001**	-0.02 [-0.15 0.12] p>0.3	1

Supplementary Table 7. Intercorrelations of spatial correspondences between sleep and cortical thickness. *Cross-sample replication of spatial distribution from phenotypic and genetic correlational analysis in F1 and FS1. ** indicates FDRq<0.05, controlled for number of tests (6) and number of markers studied (2), * indicates trend at p<0.05.*

**Phenotypical correlation between sleep duration and cortical thickness (Fig. 1B, HCP unrelated)**			
**Left superior frontal gyrus h2 = 0.46 (0.05)**			
Sample	***Depression***	***BMI***	***IQ***
HCP (unrelated sample)	0.02 [-0.07 0.12]	0.09 [-0.01 0.17]	-0.04 [-0.15 0.05]
eNKI	-0.04 [-0.13 0.05]	-0.01 [-0.09 0.05]	-0.08 [-0.15 -0.01], p<0.05*
HCP (total sample)	0.05 [-0.00 0.11]	0.04 [-0.01 0.10]	-0.11 [-0.17 -0.05], p<0.0005**
Genetic	-0.00 (0.13)	0.05 (0.08)	0.19 (0.08), p<0.05*
Enviorment	0.11 (0.06)	0.04 (0.07)	0.04 (0.07)
**Phenotypical correlation between sleep duration and cortical thickness, (Fig. 1, eNKI)**			
**Left inferior temporal cortex h2 = 0.17 (0.06)**			
Sample	***Depression***	***BMI***	***IQ***
HCP (unrelated sample)	-0.08 [-0.18 0.01]	0.02 [-0.07 0.12]	-0.01 [-0.11 0.09]
eNKI	-0.02 [-0.12 0.07]	-0.06 [-0.13 0.01]	-0.03 [-0.10 0.04]
HCP (total sample)	-0.07 [-0.13 0.00], p<0.05*	0.02 [-0.04 0.08]	0.01 [-0.05 0.07]
Genetic	0.001 (0.22)	0.27 (0.14), p<0.05*	-0.002 (0.12)
Environment	-0.07 (0.06)	-0.13 (0.07), p<0.05*	0.007 (0.06)
**Right occipital cortex h2 = 0.51 (0.05)**			
Sample	***Depression***	***BMI***	***IQ***
HCP (unrelated sample)	-0.06 [-0.15 0.04]	-0.02 [-0.12 0.08]	-0.02 [-0.12 0.08]
eNKI	-0.03 [-0.11 0.05]	0.07 [0.00 0.14], p<0.05*	-0.02 [-0.09 0.05]

HCP (total sample)	-0.03 [-0.08 0.03]	-0.00 [-0.06 0.06]	0.06 [-0.01 0.12]
Genetic	-0.03 (0.13)	0.01 (0.07)	0.19 (0.07), p<0.05*
Environment	-0.04 (0.06)	-0.02 (0.07)	-0.15 (0.07), p<0.05*
**Right inferior temporal cortex h2 = 0.29 (0.06)**			
Sample	***Depression***	***BMI***	***IQ***
HCP (unrelated sample)	-0.05 [-0.14 0.05]	0.08 [-0.01 0.18]	-0.01 [-0.10 0.08]
eNKI	-0.02 [-0.11 0.07]	-0.08 [-0.14 -0.01], p<0.05*	0.12 [0.05 0.19], p<0.0005**
HCP (total sample)	-0.01 [-0.07 0.05]	0.01 [-0.05 0.07]	0.02 [-0.04 0.08]
Genetic	0.08 (0.17)	0.10 (0.10)	0.20 (0.09), p<0.05*
Environment	-0.02 (0.06)	-0.08 (0.07)	-0.07 (0.06)

Supplementary Table 8. Intelligence and sleep duration both have phenotypic and genetic correlation with frontal regions. *Cross-sample analysis of regions associated with sleep duration on the one hand (based on F1), and depression, BMI, and IQ on the other. Please see for exact n's per analysis sample Table 4-6. ** indicates FDRq<0.05, controlled for number of samples (3), number of phenotypes (3), and number of ROIs studied (3), * indicates trend at p<0.05.*

**ROIS**	**Heritability**	**se**	**p**
'17Networks_LH_VisCent_ExStr_1'	0.3846	0.0531	0.0000
'17Networks_LH_VisCent_ExStr_2'	0.5072	0.0481	0.0000
'17Networks_LH_VisCent_ExStr_3'	0.4820	0.0494	0.0000
'17Networks_LH_VisCent_ExStr_4'	0.4852	0.0457	0.0000
'17Networks_LH_VisCent_ExStr_5'	0.3735	0.0527	0.0000
'17Networks_LH_VisCent_ExStr_6'	0.4424	0.0500	0.0000
'17Networks_LH_VisPeri_ExStrSup_1'	0.3922	0.0498	0.0000
'17Networks_LH_VisPeri_ExStrSup_2'	0.4478	0.0453	0.0000
'17Networks_LH_VisPeri_ExStrSup_3'	0.3999	0.0523	0.0000
'17Networks_LH_VisPeri_ExStrSup_4'	0.5705	0.0422	0.0000
'17Networks_LH_VisPeri_ExStrSup_5'	0.4897	0.0487	0.0000
'17Networks_LH_VisPeri_ExStrSup_6'	0.5543	0.0446	0.0000
'17Networks_LH_SomMotA_1'	0.2734	0.0598	0.0000
'17Networks_LH_SomMotA_2'	0.3185	0.0546	0.0000
'17Networks_LH_SomMotA_3'	0.4460	0.0489	0.0000
'17Networks_LH_SomMotA_4'	0.4541	0.0539	0.0000
'17Networks_LH_SomMotA_5'	0.3883	0.0547	0.0000
'17Networks_LH_SomMotA_6'	0.3030	0.0511	0.0000
'17Networks_LH_SomMotA_7'	0.4751	0.0472	0.0000
'17Networks_LH_SomMotA_8'	0.3399	0.0568	0.0000
'17Networks_LH_SomMotB_Aud_1'	0.4302	0.0550	0.0000
'17Networks_LH_SomMotB_Aud_2'	0.2452	0.0544	0.0000
'17Networks_LH_SomMotB_Aud_3'	0.4534	0.0515	0.0000
'17Networks_LH_SomMotB_Aud_4'	0.3878	0.0515	0.0000
'17Networks_LH_SomMotB_Aud_5'	0.1217	0.0576	0.0147

'17Networks_LH_SomMotB_Aud_6'	0.2482	0.0541	0.0000
'17Networks_LH_SomMotB_Aud_7'	0.5321	0.0442	0.0000
'17Networks_LH_SomMotB_Aud_8'	0.3148	0.0551	0.0000
'17Networks_LH_DorsAttnA_TempOcc_1'	0.3670	0.0585	0.0000
'17Networks_LH_DorsAttnA_TempOcc_2'	0.1722	0.0565	0.0008
'17Networks_LH_DorsAttnA_TempOcc_3'	0.2337	0.0531	0.0000
'17Networks_LH_DorsAttnA_SPL_1'	0.3574	0.0554	0.0000
'17Networks_LH_DorsAttnA_SPL_2'	0.4402	0.0539	0.0000
'17Networks_LH_DorsAttnA_SPL_3'	0.2753	0.0544	0.0000
'17Networks_LH_DorsAttnB_PostC_1'	0.2932	0.0544	0.0000
'17Networks_LH_DorsAttnB_PostC_2'	0.1653	0.0532	0.0007
'17Networks_LH_DorsAttnB_PostC_3'	0.2193	0.0545	0.0000
'17Networks_LH_DorsAttnB_PostC_4'	0.4897	0.0528	0.0000
'17Networks_LH_DorsAttnB_FEF_1'	0.2881	0.0559	0.0000
'17Networks_LH_SalVentAttnA_ParOper_1'	0.2890	0.0524	0.0000
'17Networks_LH_SalVentAttnA_Ins_1'	0.3376	0.0548	0.0000
'17Networks_LH_SalVentAttnA_Ins_2'	0.3980	0.0528	0.0000
'17Networks_LH_SalVentAttnA_Ins_3'	0.2435	0.0621	0.0000
'17Networks_LH_SalVentAttnA_ParMed_1'	0.3732	0.0585	0.0000
'17Networks_LH_SalVentAttnA_FrMed_1'	0.3584	0.0585	0.0000
'17Networks_LH_SalVentAttnA_FrMed_2'	0.2649	0.0553	0.0000
'17Networks_LH_SalVentAttnB_IPL_1'	0.2552	0.0553	0.0000
'17Networks_LH_SalVentAttnB_PFCl_1'	0.4330	0.0472	0.0000
'17Networks_LH_SalVentAttnB_PFCv_1'	0.3929	0.0533	0.0000
'17Networks_LH_SalVentAttnB_PFCmp_1'	0.3543	0.0590	0.0000
'17Networks_LH_Limbic_OFC_1'	0.2986	0.0564	0.0000
'17Networks_LH_Limbic_OFC_2'	0.4266	0.0546	0.0000
'17Networks_LH_Limbic_TempPole_1'	0.4474	0.0565	0.0000
'17Networks_LH_Limbic_TempPole_2'	0.3431	0.0497	0.0000
'17Networks_LH_Limbic_TempPole_3'	0.2563	0.0536	0.0000
'17Networks_LH_Limbic_TempPole_4'	0.4023	0.0535	0.0000
'17Networks_LH_ContA_Temp_1'	0.2404	0.0554	0.0000
'17Networks_LH_ContA_IPS_1'	0.2688	0.0541	0.0000
'17Networks_LH_ContA_IPS_2'	0.1181	0.0557	0.0146
'17Networks_LH_ContA_IPS_3'	0.2056	0.0585	0.0002
'17Networks_LH_ContA_PFCd_1'	0.3657	0.0580	0.0000
'17Networks_LH_ContA_PFCl_1'	0.2933	0.0552	0.0000
'17Networks_LH_ContA_PFCl_2'	0.3846	0.0505	0.0000
'17Networks_LH_ContA_PFCl_3'	0.2741	0.0583	0.0000
'17Networks_LH_ContA_PFCl_4'	0.2282	0.0562	0.0000
'17Networks_LH_ContA_Cinga_1'	0.4102	0.0567	0.0000
'17Networks_LH_ContB_Temp_1'	0.2019	0.0575	0.0002

'17Networks_LH_ContB_IPL_1'	0.2485	0.0571	0.0000
'17Networks_LH_ContB_PFCl_1'	0.4478	0.0488	0.0000
'17Networks_LH_ContB_PFClv_1'	0.2902	0.0568	0.0000
'17Networks_LH_ContB_PFClv_2'	0.4154	0.0522	0.0000
'17Networks_LH_ContC_pCun_1'	0.3559	0.0541	0.0000
'17Networks_LH_ContC_pCun_2'	0.4708	0.0493	0.0000
'17Networks_LH_ContC_Cingp_1'	0.5003	0.0504	0.0000
'17Networks_LH_DefaultA_IPL_1'	0.2398	0.0530	0.0000
'17Networks_LH_DefaultA_PFCd_1'	0.4703	0.0481	0.0000
'17Networks_LH_DefaultA_PCC_1'	0.2171	0.0534	0.0000
'17Networks_LH_DefaultA_PCC_2'	0.4264	0.0531	0.0000
'17Networks_LH_DefaultA_PCC_3'	0.3635	0.0559	0.0000
'17Networks_LH_DefaultA_PFCm_1'	0.3291	0.0568	0.0000
'17Networks_LH_DefaultA_PFCm_2'	0.4869	0.0471	0.0000
'17Networks_LH_DefaultA_PFCm_3'	0.4891	0.0470	0.0000
'17Networks_LH_DefaultB_Temp_1'	0.2430	0.0593	0.0000
'17Networks_LH_DefaultB_Temp_2'	0.2888	0.0559	0.0000
'17Networks_LH_DefaultB_Temp_3'	0.3364	0.0575	0.0000
'17Networks_LH_DefaultB_Temp_4'	0.2035	0.0579	0.0002
'17Networks_LH_DefaultB_IPL_1'	0.2323	0.0604	0.0000
'17Networks_LH_DefaultB_PFCd_1'	0.4882	0.0481	0.0000
'17Networks_LH_DefaultB_PFCd_2'	0.5197	0.0461	0.0000
'17Networks_LH_DefaultB_PFCd_3'	0.3488	0.0547	0.0000
'17Networks_LH_DefaultB_PFCd_4'	0.4556	0.0507	0.0000
'17Networks_LH_DefaultB_PFCv_1'	0.3230	0.0511	0.0000
'17Networks_LH_DefaultB_PFCv_2'	0.2887	0.0516	0.0000
'17Networks_LH_DefaultB_PFCv_3'	0.1730	0.0551	0.0006
'17Networks_LH_DefaultB_PFCv_4'	0.3490	0.0573	0.0000
'17Networks_LH_DefaultC_IPL_1'	0.3328	0.0564	0.0000
'17Networks_LH_DefaultC_Rsp_1'	0.4252	0.0540	0.0000
'17Networks_LH_DefaultC_PHC_1'	0.4522	0.0516	0.0000
'17Networks_LH_TempPar_1'	0.2145	0.0606	0.0001
'17Networks_LH_TempPar_2'	0.3099	0.0547	0.0000
'17Networks_RH_VisCent_ExStr_1'	0.4343	0.0533	0.0000
'17Networks_RH_VisCent_ExStr_2'	0.2609	0.0542	0.0000
'17Networks_RH_VisCent_ExStr_3'	0.4995	0.0508	0.0000
'17Networks_RH_VisCent_ExStr_4'	0.4546	0.0516	0.0000
'17Networks_RH_VisCent_ExStr_5'	0.2379	0.0531	0.0000
'17Networks_RH_VisCent_ExStr_6'	0.5103	0.0485	0.0000
'17Networks_RH_VisPeri_ExStrSup_1'	0.5629	0.0438	0.0000
'17Networks_RH_VisPeri_ExStrSup_2'	0.5330	0.0482	0.0000
'17Networks_RH_VisPeri_ExStrSup_3'	0.5804	0.0444	0.0000

'17Networks_RH_VisPeri_ExStrSup_4'	0.5045	0.0499	0.0000
'17Networks_RH_VisPeri_ExStrSup_5'	0.4991	0.0467	0.0000
'17Networks_RH_VisPeri_ExStrSup_6'	0.3547	0.0531	0.0000
'17Networks_RH_SomMotA_1'	0.3411	0.0592	0.0000
'17Networks_RH_SomMotA_2'	0.1940	0.0588	0.0003
'17Networks_RH_SomMotA_3'	0.3230	0.0524	0.0000
'17Networks_RH_SomMotA_4'	0.4289	0.0513	0.0000
'17Networks_RH_SomMotA_5'	0.2538	0.0538	0.0000
'17Networks_RH_SomMotA_6'	0.4673	0.0516	0.0000
'17Networks_RH_SomMotA_7'	0.3577	0.0582	0.0000
'17Networks_RH_SomMotA_8'	0.2417	0.0586	0.0000
'17Networks_RH_SomMotA_9'	0.4900	0.0470	0.0000
'17Networks_RH_SomMotA_10'	0.3734	0.0504	0.0000
'17Networks_RH_SomMotA_11'	0.3822	0.0533	0.0000
'17Networks_RH_SomMotB_S2_1'	0.4087	0.0530	0.0000
'17Networks_RH_SomMotB_S2_2'	0.2605	0.0615	0.0000
'17Networks_RH_SomMotB_S2_3'	0.3401	0.0567	0.0000
'17Networks_RH_SomMotB_S2_4'	0.2867	0.0572	0.0000
'17Networks_RH_SomMotB_S2_5'	0.1916	0.0574	0.0003
'17Networks_RH_SomMotB_S2_6'	0.2819	0.0579	0.0000
'17Networks_RH_SomMotB_S2_7'	0.4855	0.0497	0.0000
'17Networks_RH_DorsAttnA_TempOcc_1'	0.2867	0.0563	0.0000
'17Networks_RH_DorsAttnA_TempOcc_2'	0.1993	0.0566	0.0002
'17Networks_RH_DorsAttnA_SPL_1'	0.3348	0.0539	0.0000
'17Networks_RH_DorsAttnA_SPL_2'	0.3804	0.0550	0.0000
'17Networks_RH_DorsAttnA_SPL_3'	0.1553	0.0536	0.0014
'17Networks_RH_DorsAttnA_SPL_4'	0.4021	0.0534	0.0000
'17Networks_RH_DorsAttnB_PostC_1'	0.2626	0.0532	0.0000
'17Networks_RH_DorsAttnB_PostC_2'	0.1845	0.0584	0.0005
'17Networks_RH_DorsAttnB_PostC_3'	0.3525	0.0535	0.0000
'17Networks_RH_DorsAttnB_PostC_4'	0.3309	0.0515	0.0000
'17Networks_RH_DorsAttnB_FEF_1'	0.3419	0.0578	0.0000
'17Networks_RH_SalVentAttnA_ParOper_1'	0.2819	0.0527	0.0000
'17Networks_RH_SalVentAttnA_PrC_1'	0.1674	0.0595	0.0017
'17Networks_RH_SalVentAttnA_Ins_1'	0.3765	0.0535	0.0000
'17Networks_RH_SalVentAttnA_Ins_2'	0.4515	0.0569	0.0000
'17Networks_RH_SalVentAttnA_Ins_3'	0.2235	0.0580	0.0000
'17Networks_RH_SalVentAttnA_FrMed_1'	0.2194	0.0554	0.0000
'17Networks_RH_SalVentAttnA_FrMed_2'	0.2607	0.0580	0.0000
'17Networks_RH_SalVentAttnA_FrMed_3'	0.4393	0.0534	0.0000
'17Networks_RH_SalVentAttnA_FrMed_4'	0.3503	0.0579	0.0000
'17Networks_RH_SalVentAttnB_IPL_1'	0.1638	0.0542	0.0008

'17Networks_RH_SalVentAttnB_PFCl_1'	0.3806	0.0525	0.0000
'17Networks_RH_SalVentAttnB_PFCl_2'	0.3708	0.0521	0.0000
'17Networks_RH_SalVentAttnB_PFCv_1'	0.2348	0.0561	0.0000
'17Networks_RH_SalVentAttnB_PFCv_2'	0.3004	0.0536	0.0000
'17Networks_RH_SalVentAttnB_PFCmp_1'	0.3030	0.0557	0.0000
'17Networks_RH_Limbic_OFC_1'	0.4312	0.0535	0.0000
'17Networks_RH_Limbic_OFC_2'	0.2843	0.0528	0.0000
'17Networks_RH_Limbic_OFC_3'	0.3339	0.0528	0.0000
'17Networks_RH_Limbic_OFC_4'	0.3922	0.0531	0.0000
'17Networks_RH_Limbic_TempPole_1'	0.2574	0.0571	0.0000
'17Networks_RH_Limbic_TempPole_2'	0.3366	0.0543	0.0000
'17Networks_RH_Limbic_TempPole_3'	0.4622	0.0503	0.0000
'17Networks_RH_Limbic_TempPole_4'	0.3670	0.0503	0.0000
'17Networks_RH_ContA_IPS_1'	0.1667	0.0579	0.0017
'17Networks_RH_ContA_IPS_2'	0.1824	0.0580	0.0006
'17Networks_RH_ContA_PFCd_1'	0.2794	0.0565	0.0000
'17Networks_RH_ContA_PFCl_1'	0.2659	0.0542	0.0000
'17Networks_RH_ContA_PFCl_2'	0.4317	0.0512	0.0000
'17Networks_RH_ContA_Cinga_1'	0.3963	0.0560	0.0000
'17Networks_RH_ContB_Temp_1'	0.3178	0.0564	0.0000
'17Networks_RH_ContB_Temp_2'	0.2546	0.0599	0.0000
'17Networks_RH_ContB_IPL_1'	0.1730	0.0548	0.0006
'17Networks_RH_ContB_IPL_2'	0.1359	0.0529	0.0043
'17Networks_RH_ContB_PFCld_1'	0.3162	0.0520	0.0000
'17Networks_RH_ContB_PFCld_2'	0.3238	0.0559	0.0000
'17Networks_RH_ContB_PFClv_1'	0.2573	0.0552	0.0000
'17Networks_RH_ContB_PFClv_2'	0.3330	0.0535	0.0000
'17Networks_RH_ContB_PFCmp_1'	0.4738	0.0524	0.0000
'17Networks_RH_ContB_PFCmp_2'	0.2798	0.0555	0.0000
'17Networks_RH_ContC_pCun_1'	0.2809	0.0528	0.0000
'17Networks_RH_ContC_pCun_2'	0.2339	0.0536	0.0000
'17Networks_RH_ContC_Cingp_1'	0.4953	0.0507	0.0000
'17Networks_RH_DefaultA_IPL_1'	0.1987	0.0584	0.0002
'17Networks_RH_DefaultA_PFCd_1'	0.3935	0.0520	0.0000
'17Networks_RH_DefaultA_PCC_1'	0.4024	0.0536	0.0000
'17Networks_RH_DefaultA_PFCm_1'	0.2954	0.0536	0.0000
'17Networks_RH_DefaultA_PFCm_2'	0.3168	0.0580	0.0000
'17Networks_RH_DefaultA_PFCm_3'	0.4968	0.0494	0.0000
'17Networks_RH_DefaultB_Temp_1'	0.2870	0.0551	0.0000
'17Networks_RH_DefaultB_AntTemp_1'	0.3025	0.0544	0.0000
'17Networks_RH_DefaultB_PFCd_1'	0.4688	0.0475	0.0000
'17Networks_RH_DefaultB_PFCv_1'	0.3612	0.0536	0.0000

'17Networks_RH_DefaultC_IPL_1'	0.1649	0.0579	0.0016
'17Networks_RH_DefaultC_Rsp_1'	0.3930	0.0545	0.0000
'17Networks_RH_DefaultC_PHC_1'	0.4144	0.0544	0.0000
'17Networks_RH_TempPar_1'	0.4116	0.0572	0.0000
'17Networks_RH_TempPar_2'	0.2234	0.0584	0.0000
'17Networks_RH_TempPar_3'	0.2932	0.0602	0.0000
'17Networks_RH_TempPar_4'	0.2303	0.0560	0.0000

Supplementary Table 9. Parcel-wise heritability of cortical thickness in HCP. *Heritability, standard error and p-values of cortical thickness controlling for global thickness, as well as age, sex, age*age, and age*sex using Solar 8.4.0. All regions were heritable at FDRq<0.05.*

Confound	Environmental correlation	Genetic correlation
**Depression**		
Left superior frontal gyrus	0.12(0.06), p>0.05.	-0.46(0.12), p<0.0002
Right superior frontal gyrus	0.15(0.06), p<0.02	-0.46(0.13), p<0.0002
**BMI**		
Left superior frontal gyrus	0.11(0.06), p>0.05	-0.45(0.13), p<0.0004
Right superior frontal gyrus	0.13(0.06), p<0.05	-0.45(0.14), p<0.0005
**Intelligence**		
Left superior frontal gyrus	0.09(0.06), p=n.s.	-0.41(0.13), p<0.002
Right superior frontal gyrus	0.09(0.06), p=n.s.	-0.39(0.14), p<0.004

Supplementary Table 10. Post-hoc test for potential confounds (depression/BMI/intelligence) on genetic correlation between duration of sleep and cortical thickness. *Environmental and genetic correlation as well as std. error are displayed.*

**Genetic correlation (based on whole brain analysis in HCP total sample)**		
Left superior frontal gyrus	r	p
HCP (unrelated sample)	-0.12	0.014*
eNKI	-0.02	0.64
HCP (total sample) (n=1113)	-0.07	0.016*
Genetic correlation	-0.46	0.0001**
Environmental correlation	0.12	0.0503
Right superior frontal gyrus	r	p
HCP (unrelated sample)	-0.08	0.11
eNKI	-0.02	0.70
HCP (total sample) (n=1113)	-0.06	0.03*
Genetic correlation	-0.46	0.0002**
Enviromental correlation	0.15	0.014*

Supplementary Table 11. Genetic correlations between sleep duration and cortical thickness were inconsistent across HCP and eNKI samples. *Cross-sample replication of FDR-corrected ROIs from genetic correlation analysis in Supplementary Fig. 1. ** indicates significant at whole brain level (FDRq<0.05/2), * indicates trending correlation (p<0.05).*

References:

1 Schaefer, A. *et al.* Local-Global Parcellation of the Human Cerebral Cortex from Intrinsic Functional Connectivity MRI. *Cereb Cortex* **28**, 3095-3114, doi:10.1093/cercor/bhx179 (2018).

2 Fox, P. T., Lancaster, J. L., Laird, A. R. & Eickhoff, S. B. Meta-analysis in human neuroimaging: computational modeling of large-scale databases. *Annu Rev Neurosci* **37**, 409-434, doi:10.1146/annurev-neuro-062012-170320 (2014).

3 Glasser, M. F. *et al.* The minimal preprocessing pipelines for the Human Connectome Project. *Neuroimage* **80**, 105-124, doi:10.1016/j.neuroimage.2013.04.127 (2013).

4 Dashti, H. S. *et al.* Genome-wide association study identifies genetic loci for self-reported habitual sleep duration supported by accelerometer-derived estimates. *Nature Communications* **10**, 1100, doi:10.1038/s41467-019-08917-4 (2019).
