# Supplementary material for: The interrelation of sleep and mental and physical health is anchored in grey-matter neuroanatomy and under genetic control
Source: Commun Biol. 2020 Apr 9;3:171. doi: 10.1038/s42003-020-0892-6 (PMC7145855; doi:10.1038/s42003-020-0892-6)
Supplement: Supplementary file 3 — Reporting Summary [file 42003_2020_892_MOESM3_ESM.pdf]

nature research

Corresponding author(s): Sofie Valk

Last updated by author(s): Mar 10, 2020

Reporting Summary

Nature Research wishes to improve the reproducibility of the work that we publish. This form provides structure for consistency and transparency in reporting. For further information on Nature Research policies, see Authors & Referees and the Editorial Policy Checklist.

Statistics

For all statistical analyses, confirm that the following items are present in the figure legend, table legend, main text, or Methods section.

n/a	Confirmed	
☐	☒	The exact sample size (*n*) for each experimental group/condition, given as a discrete number and unit of measurement
☐	☒	A statement on whether measurements were taken from distinct samples or whether the same sample was measured repeatedly
☐	☒	The statistical test(s) used AND whether they are one- or two-sided<br>*Only common tests should be described solely by name; describe more complex techniques in the Methods section.*
☐	☒	A description of all covariates tested
☐	☒	A description of any assumptions or corrections, such as tests of normality and adjustment for multiple comparisons
☐	☒	A full description of the statistical parameters including central tendency (e.g. means) or other basic estimates (e.g. regression coefficient) AND variation (e.g. standard deviation) or associated estimates of uncertainty (e.g. confidence intervals)
☐	☒	For null hypothesis testing, the test statistic (e.g. *F*, *t*, *r*) with confidence intervals, effect sizes, degrees of freedom and *P* value noted<br>*Give P values as exact values whenever suitable.*
☒	☐	For Bayesian analysis, information on the choice of priors and Markov chain Monte Carlo settings
☒	☐	For hierarchical and complex designs, identification of the appropriate level for tests and full reporting of outcomes
☒	☐	Estimates of effect sizes (e.g. Cohen's *d*, Pearson's *r*), indicating how they were calculated

Our web collection on statistics for biologists contains articles on many of the points above.

Software and code

Policy information about availability of computer code

Data collection	All data analyzed in this manuscript were obtained from the open-access HCP young adult sample (HCP; http://www.humanconnectome.org/) and enhanced NKI-Rockland sample (https://www.ncbi.nlm.nih.gov/pmc/articles/PMC3472598/). MRI images were acquired from the International Neuroimaging Data Sharing Initiative (INDI) online database http://fcon_1000.projects.nitrc.org/indi/enhanced/studies.html.
Data analysis	Phenotypic analysis were all performed in Matlab version 2017a using standard statistical toolboxes. Cortical thickness measures were derived via HCP's Freesurfer protocol, and Freesurfer 6.0 (eNKI) and extracted in 400 parcels. We studied sleep quantity (self-reported sleep duration) and global sleep quality (total PSQI) score using the Pittsburg Sleep Quality Index (PSQI), and also extracted BMI, intelligence, and depression score from HCP and eNKI databases to identify correlation between sleep parameters and physical and mental scores. Solar eclipse 8.4.0. (http://solar-eclipse-genetics.org) was used to perform heritability analysis and to create a matrix of genetic correlation between local thickness. Subsequently, we performed partial least squares (PLS) analysis to identify latent relationships between the mentioned factors.

For manuscripts utilizing custom algorithms or software that are central to the research but not yet described in published literature, software must be made available to editors/reviewers. We strongly encourage code deposition in a community repository (e.g. GitHub). See the Nature Research guidelines for submitting code & software for further information.

Data

Policy information about availability of data

All manuscripts must include a data availability statement. This statement should provide the following information, where applicable:

- Accession codes, unique identifiers, or web links for publicly available datasets
- A list of figures that have associated raw data
- A description of any restrictions on data availability

All data analyzed in this manuscript were obtained from the open-access HCP young adult sample (HCP; http://www.humanconnectome.org/)(Van Essen, Smith et al. 2013) and enhanced NKI-Rockland sample (https://www.ncbi.nlm.nih.gov/pmc/articles/PMC3472598/) (Nooner, Colcombe et al. 2012). Scans were acquired

from the International Neuroimaging Data Sharing Initiative (INDI) online database http://fcon_1000.projects.nitrc.org/indi/enhanced/studies.html. The raw data may not be shared by third parties due to ethics requirements, but can be downloaded directly via the above weblinks. Spearman correlations and confidence intervals were computed using the Robust Correlation toolbox https://github.com/CPernet/robustcorrtool (Pernet, Wilcox et al. 2012)
Genetic analyses were performed using Solar Eclipse 8.4.0 (http://www.solar-eclipse-genetics.org), and data on the KING pedigree analysis is available here: https://www.nitrc.org/projects/se_linux/ (Almasy and Blangero 1998, Kochunov, Donohue et al. 2019). We performed partial least square analysis using https://miplab.epfl.ch/index.php/software/PLS. (McIntosh and Lobaugh 2004, Zoller, Schaer et al. 2017). Brainmap analysis were performed using http://www.brainmap.org(Laird, Eickhoff et al. 2009, Laird, Eickhoff et al. 2011). Main analysis scripts and genetic correlation tables are available in https://github.com/sofievalk/projects/tree/master/Tahmasian_Sleep.

Field-specific reporting

Please select the one below that is the best fit for your research. If you are not sure, read the appropriate sections before making your selection.

☒ Life sciences ☐ Behavioural & social sciences ☐ Ecological, evolutionary & environmental sciences

For a reference copy of the document with all sections, see nature.com/documents/nr-reporting-summary-flat.pdf

Life sciences study design

All studies must disclose on these points even when the disclosure is negative.

Sample size	We studied two independent samples from openly-shared neuroimaging repositories, Human Connectome Project and enhanced eNKI. Human Connectome Project (HCP; http://www.humanconnectome.org/), comprised data from 1105 individuals (599 females), 285 MZ twins, 170 DZ twins, and 650 singletons, with mean age 28.8 years (SD = 3.7, range = 22–37). For phenotypic analysis we selected unrelated individuals resulting in a sample of 424 (228 females) individuals with mean age of 28.6 years (SD =3.7, range =22-36). Our second sample was based on the Nathan Kline Institute-Rockland Sample (eNKI), made available by the Nathan-Kline Institute (NKY, NY, USA). This sample consisted of 783 (487 females) individuals with mean age of 41.2 years (SD =20.3, range =12-85) enabling us to identify life-span relations between sleep, brain structure and behaviour.
Data exclusions	We included participants for whom the MRI images and data had been released (humanconnectome.org) after passing the HCP quality control and assurance standards. The full set of inclusion and exclusion criteria are described previously (Marcus, 2013).<br>For our phenotypical analyses in eNKI database, we selected individuals with complete sleep and imaging data.
Replication	We used Nathan Kline Institute-Rockland Sample (eNKI), made available by the Nathan-Kline Institute (NKY, NY, USA) to replicate our results in HCP.
Randomization	No randomization is performed.
Blinding	No blinding is done.

Reporting for specific materials, systems and methods

We require information from authors about some types of materials, experimental systems and methods used in many studies. Here, indicate whether each material, system or method listed is relevant to your study. If you are not sure if a list item applies to your research, read the appropriate section before selecting a response.

Materials & experimental systems

n/a	Involved in the study	
☒	☐	Antibodies
☒	☐	Eukaryotic cell lines
☒	☐	Palaeontology
☒	☐	Animals and other organisms
☐	☒	Human research participants
☒	☐	Clinical data

Methods

n/a	Involved in the study	
☒	☐	ChIP-seq
☒	☐	Flow cytometry
☐	☒	MRI-based neuroimaging

Human research participants

Policy information about studies involving human research participants

Population characteristics	HCP: For our phenotypic analyses, we selected an unrelated subsample with complete behavioral data (n=457). After removing individuals with missing structural imaging our sample for phenotypiccorrelations consisted of 424 (228 females) individuals with mean age of 28.6 years (SD =3.7, range =22-36). For our twin-based genetic analyses, we used the complete sample of individuals with complete structural imaging for structural gray matter and behavioral data for sleep genetic correlation analyses including 1105 individuals (599 females), 285 MZ twins, 170 DZ twins, and 650 singletons, with mean age 28.8 years (SD = 3.7,

range = 22–37).
eNKI: Our sample for phenotypical correlations consisted of 783 (487 females) individuals with mean age of 41.2 years (SD =20.3, range =12-85).

Recruitment

The full set of inclusion and exclusion criteria are described previously (Marcus, 2013). https://www.ncbi.nlm.nih.gov/pubmed/23707591

Ethics oversight

Heinrich-Heine-Universitaet Duesseldorf

Note that full information on the approval of the study protocol must also be provided in the manuscript.

Magnetic resonance imaging

Experimental design

Design type

Structural (T1) imaging

Design specifications

not relevant

Behavioral performance measures

HCP: First, to constrain analyses we selected primary markers for cognition, mental and physical health based on the relation of sleep to these traits in HCP. The selected traits include 38 emotional, cognitive, NEO-FFI personality, as well as the 7 PSQI sleep markers for reference, based on the unrestricted phenotypic data as well as 46 mental and physical health markers based on the restricted phenotypic data. For more information on available phenotypes, please see: (https://wiki.humanconnectome.org/display/PublicData).
Inter-individual difference in sleep quality were derived from information of the self-reported Pittsburg Sleep Questionnaire (PSQI) (Buysse, 1989) , which is common measure of sleep quality with significant item-level reliability and validity. For markers of life function, we used BMI (703 * weight/ (height)^2) and the ASR depression DSM-oriented scale for Ages 18-5949 (https://aseba.org/). As a proxy for intelligence we used the NIH Toolbox Cognition Weintraub, 2013 , 'total composite score'.
eNKI: Sleep markers were derived from the Pittsburg Sleep Questionnaire (see further the section on this question in the HCP sample). Intelligence was measured using the Wechsler Abbreviated Scale of Intelligence (WASI-II). Depression was measured using the Beck Depression Inventory (BDI – II) and Body-mass-index was calculated using weight and height. These vitals are obtained and recorded by study staff. Height was recorded in centimeters. Weight was recorded in kilograms. Body Mass Index (BMI) was automatically calculated.

Acquisition

Imaging type(s)

Structural imaging

Field strength

3 Tesla

Sequence & imaging parameters

3D magnetization-prepared rapid gradient-echo imaging (3D MP-RAGE) structural scans93 were acquired using a 3.0T Siemens Trio scanner with TR=2500ms, TE=3.5 ms, Bandwidth=190 Hz/Px, FoV=256 × 256 mm, flip angle=8°, voxel size=1.0 × 1.0 × 1.0 mm. More details on image acquisition are available at http://fcon_1000.projects.nitrc.org/indi/enhanced/studies.html and also described previously (Marcus, 2013).

Area of acquisition

Whole brain

Diffusion MRI ☐ Used ☒ Not used

Preprocessing

Preprocessing software

All T1 scans were visually inspected to ensure the absence of gross artefacts and subsequently pre-processed using the Freesurfer software library (http://surfer.nmr.mgh.harvard.edu/), version 5.3.0 (Fischl, 2012) using the standard HCP-Freesurfer protocol.

Normalization

using the standard HCP-Freesurfer protocol

Normalization template

MNI

Noise and artifact removal

using the standard HCP-Freesurfer protocol.

Volume censoring

n/a

Statistical modeling & inference

Model type and settings

Phenotypic analysis: Here we computed Spearman correlations, controlling for age, sex, age * age, sex * age, and in case of imaging data global thickness.
Genetic correlation analysis: Within SOLAR, this is assessed by contrasting the observed covariance matrices for a phenotypic (neuroimaging or behavioral) measure with the structure of the covariance matrix predicted by kinship. Heritability analyses were conducted with simultaneous estimation for the effects of potential covariates. For this study,

we included covariates including age, sex, age × sex interaction, age2, age2 × sex interaction. To determine if variations in sleep and brain structure were influenced by the same genetic factors, genetic correlation analyses were conducted. Specifically, bivariate polygenic analyses were performed to estimate genetic (ρg) and environmental (ρe) correlations, based on the phenotypic correlation (ρp), between brain structure and sleep with the following formula: ρp = ρg√(h21h22) + ρe√[(1 − h21)(1 − h22)], where h21 and h22 are the heritability's of the parcel-based cortical thickness and the sleep parameters. The significance of these correlations was tested by comparing the log likelihood for two restricted models (with either ρg or ρe constrained to be equal to 0) against the log likelihood for the model in which these parameters were estimated.
Partial least squares: PLS is a multivariate data-driven statistical technique that aims to maximize the covariance between two matrices by deriving latent components (LCs), which are optimal linear combinations of the original matrices 101,102. We applied PLS to the cortical thickness and sleep, BMI, depression, and IQ measures of all participants. In short, PLS performs data normalization, cross-covariance, and singular value decomposition. Following, brain and behavioral scores are created and permutation testing is performed to assess significance of each latent factor solution. Last, bootstrapping is performed to test the stability of the brain saliencies.

Effect(s) tested

Phenotypic correlation between behavioral marker and local brain structure.
Heritability of behavioral marker and local brain structure.
Genetic correlation of behavioral marker and local brain structure.
Partial least squares: PLS is a multivariate data-driven statistical technique that aims to maximize the covariance between two matrices by deriving latent components (LCs), which are optimal linear combinations of the original matrices (Zoller, 2017; McIntosh, 2004)

Specify type of analysis: ☒ Whole brain ☐ ROI-based ☐ Both

Statistic type for inference
(See Eklund et al. 2016)

Parcel-wise. We segmented brain structure in 200 parcels. Following effects were evaluated using FDR correlations (p<0.05), controlling for number of parcels.

Correction

FDR correction using number of tests

Models & analysis

n/a	Involved in the study
☒	☐ Functional and/or effective connectivity
☒	☐ Graph analysis
☒	☐ Multivariate modeling or predictive analysis
